# Supplementary material for: Antimicrobial Peptide Conjugated on Graphene Oxide-Containing Sulfonated Polyetheretherketone Substrate for Effective Antibacterial Activities against Staphylococcus aureus
Source: Antibiotics (Basel). 2023 Sep 5;12(9):1407. doi: 10.3390/antibiotics12091407 (PMC10525520; doi:10.3390/antibiotics12091407)
Supplement: Supplementary file 1 [file antibiotics-12-01407-s001.zip › antibiotics-2518607-supplementary.docx]

**Supplementary informations**

**Antimicrobial Peptide Conjugated on Graphene Oxide- Containing Sulfonated Polyetheretherketone Substrate for Effective Antibacterial Activities against *Staphylococcus aureus***

**Selvaraj Rajesh Kumar^1^, Chih-Chien Hu^2^, Truong Thi Tuong Vi^1,3^ , Dave W. Chen^4*^, and Shingjiang Jessie Lue ^1,4,5*^**

^1^ Department of Chemical and Materials Engineering, Chang Gung University, Taoyuan City 333, Taiwan; rajeshkumarcgu@mail.cgu.edu.tw (S.R.K.); vi123456@cgmh.org.tw (T.T.T.V.)

^2^ Department of Orthopedics, Chang Gung Memorial Hospital, Linkou, Taoyuan City 333, Taiwan; [r52906154@cgmh.org.tw](mailto:r52906154@cgmh.org.tw) (C.C.H)

^3^ Division of Pediatric Gastroenterology and Hepatology, Department of Pediatrics, Chang Gung Memorial Hospital, Taoyuan City 333, Taiwan

^4^ Department of Orthopedic Surgery, Chang Gung Memorial Hospital, Keelung City 204, Taiwan

^5^ Department of Safety, Health and Environment Engineering, Ming Chi University of Technology, New Taipei City 243, Taiwan

*****The statements, opinions and data contained in all publications are solely : mr5181@cgmh.org.tw (D.W.C.); jessie@mail.cgu.edu.tw (S.J.L.);
Tel.: +886-32118800 (ext. 5489) (S.J.L.); Fax: +886-32118700 (S.J.L.)

**
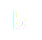

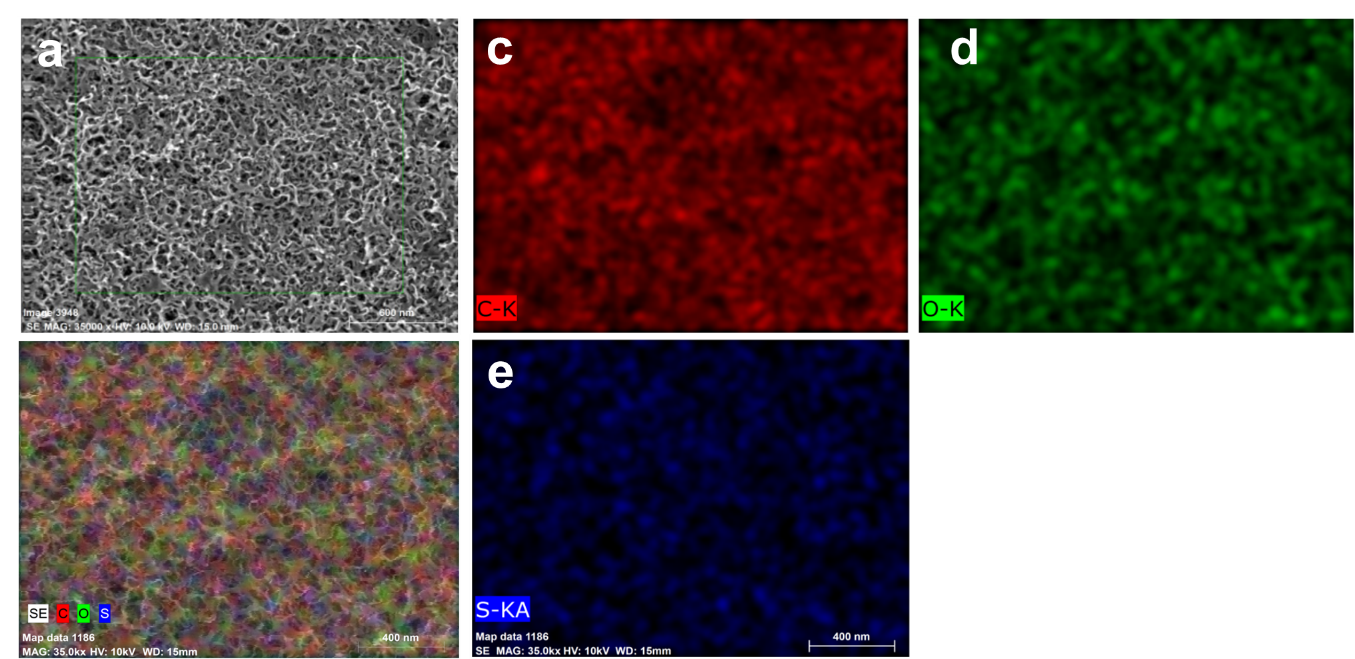
**

b

**Figure S1.** a) Full scanning area of surface microscopic image of SPEEK and energy dispersive X-ray analysis of b) full elemental mapping and detailed single elemental mapping of c) carbon, d) oxygen and e) Sulphur.

**
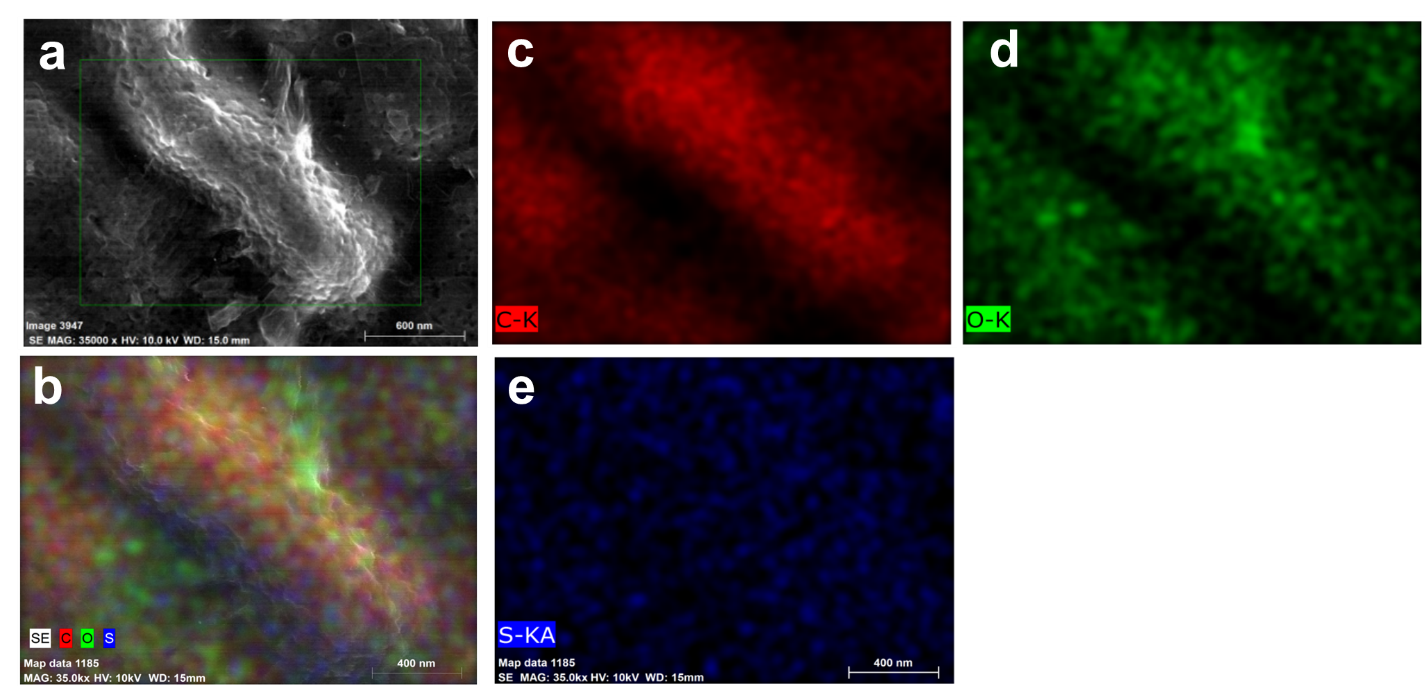
**

**Figure S2.** a) Full scanning area of surface microscopic image of SPEEK-GO and energy dispersive X-ray analysis of b) full elemental mapping and detailed single elemental mapping of c) carbon, d) oxygen and e) Sulphur.
